# Posttranscriptional Regulation of the Human ABCG2 Multidrug Transporter Protein by Artificial Mirtrons

**DOI:** 10.3390/genes12071068

**Published:** 2021-07-13

**Authors:** Anita Schamberger, György Várady, Ábel Fóthi, Tamás I. Orbán

**Affiliations:** Institute of Enzymology, ELKH Research Centre for Natural Sciences, H-1117 Budapest, Hungary; varady.gyorgy@ttk.hu (G.V.); fothi.abel@gmail.com (Á.F.)

**Keywords:** mirtron, miRNA, ABCG2, silencing, multidrug transporter

## Abstract

ABCG2 is a membrane transporter protein that has been associated with multidrug resistance phenotype and tumor development. Additionally, it is expressed in various stem cells, providing cellular protection against endobiotics and xenobiotics. In this study, we designed artificial mirtrons to regulate ABCG2 expression posttranscriptionally. Applying EGFP as a host gene, we could achieve efficient silencing not only in luciferase reporter systems but also at the ABCG2 protein level. Moreover, we observed important new sequential-functional features of the designed mirtrons. Mismatch at the first position of the mirtron-derived small RNA resulted in better silencing than full complementarity, while the investigated middle and 3′ mismatches did not enhance silencing. These latter small RNAs operated most probably via non-seed specific translational inhibition in luciferase assays. Additionally, we found that a mismatch in the first position has not, but a second mismatch in the third position has abolished target mRNA decay. Besides, one nucleotide mismatch in the seed region did not impair efficient silencing at the protein level, providing the possibility to silence targets carrying single nucleotide polymorphisms or mutations. Taken together, we believe that apart from establishing an efficient ABCG2 silencing system, our designing pipeline and results on sequential-functional features are beneficial for developing artificial mirtrons for other targets.

## 1. Introduction

The human ABCG2 protein is one of the 48 known members of the human ATP-binding-cassette (ABC) protein family. It was originally cloned from the placenta and cells selected for multidrug resistance [1,2,3], but according to our present knowledge, ABCG2 is also expressed in various differentiated tissues, including ovary, kidney, liver, breast epithelial cells, intestinal epithelia, and the blood–brain barrier [4]. This multidrug transporter protein provides resistance against various endo- and xenobiotics and hypothesized to play a physiological role in the chemoimmunity defense system [5]. The ABCG2 protein has also been identified in many types of tissue-derived stem cells and in human embryonic stem cell lines (hESC), and its role is presumably the protection against different toxins and stress [6,7]. Moreover, its expression was shown to be a reliable marker of the “side-population phenotype” [8]; therefore, investigating its role and function in various stem cells is still an important issue. There are several model systems where ABCG2 is overexpressed or knocked out [9,10,11]; however, a model where the function of ABCG2 is turned off in a carefully controlled and reversible manner is lacking. MicroRNA (miRNA)-based regulation could present a versatile platform for such purposes, providing a posttranscriptional fine-tuning of gene expression, thereby a careful studying of protein function.

The majority of miRNAs are processed via the canonical miRNA biogenesis pathway. The ~20–24 nucleotide (nt) long, single-stranded mature RNA derives from an imperfect RNA hairpin structure, which is usually transcribed from the genome by a Pol II polymerase [12,13]. This primary transcript (pri-miRNA) is then cleaved by a nuclear RNase III-like enzyme Drosha (assisted by its partner protein, DGCR8), releasing a ~60–70 nt long hairpin (called pre-miRNA; [14,15,16,17]). The pre-miRNA is then transported from the nucleus to the cytoplasm by the Exportin-5 shuttle system [18,19,20,21]. In the cytoplasm, Dicer, another RNase III-like enzyme, cleaves the pre-miRNA, liberating the double-stranded miRNA:miRNA* molecule [22,23]. Dicer acts as a molecular ruler, and the cleavage site can be measured either from the 5′- or the 3′-end of the pre-miRNA, depending on the stability of the 5′-end [24,25,26,27]. During further processing, one strand (called guide strand) of the liberated small RNA duplex is incorporated into an Argonaute (AGO) protein-containing complex, forming a mature RISC (RNA induced silencing complex) and guiding it to the target transcript. Up to our present knowledge, strand selection is mainly determined by thermodynamic characteristics (strands with low thermodynamic stability at their 5′-end are favorable) and the 5′ nucleotide identity (A and U are favorable [28]). The regulatory effect of miRNAs is usually manifested by the destabilization/degradation and/or translational inhibition of the target mRNA molecule via the partial base pairing of the miRNA and the 3′-untranslated region (3′-UTR) of the mRNA [29,30].

Non-canonical miRNA biogenesis pathways could bypass certain steps of the canonical process, typically one or even both of the two cleavage steps [31,32,33,34]. Mirtrons, which are generated in a Drosha-independent pathway, represent the most prominent group of the alternatively processed miRNAs. They reside in short introns, which are essentially equivalent to the precursor form (pre-miRNA) of the given miRNA. Thus, the first step of the mirtronic miRNA processing is different from the canonical one: the pre-miRNA is liberated from the primary transcript by the splicing machinery instead of the Drosha/DGCR8 complex. The mirtron pathway was first described in *Drosophila melanogaster* and *Caenorhabditis elegans* [35,36], and later, it was experimentally demonstrated to be operational also in mammals [37,38,39].

Mirtrons, owing to their special features, are promising genetic tools for the regulation of genes of interest. They could be expressed by Pol II promoters; therefore, their expression can be spatiotemporally regulated, while their maturation does not interfere in the nucleus with the endogenous canonical miRNA maturation pathway [37,40]. There are several articles investigating the potential of artificially designed mirtrons as silencers and showing additional advantages, such as embedding multiple artificial mirtrons in a gene for delivery and investigating various therapeutic potentials [40,41,42,43].

In this study, we present the design of artificial mirtrons for silencing the ABCG2 multidrug transporter protein. Testing several potential candidates, we could successfully silence targets in luciferase reporter assays. Moreover, we could also effectively reduce the protein level of ABCG2. In addition, we observed important sequential-functional features of the designed mirtrons. Changing the complementarity to the target in various positions revealed the importance of the middle and 3′ region in more efficient repression, while one mismatch in the first position or the seed region did not abolish efficient silencing. The various changes also influenced the balance between translation inhibition and mRNA destabilization. As an important aspect, we also point out to consider the presence of nucleotide polymorphisms when designing mirtrons against a particular gene of interest.

## 2. Materials and Methods

### 2.1. Bioinformatics, Statistical Analysis

During mirtron design, we used several prediction programs to select promising artificial mirtron sequences for further experimental investigations. After designing the different mirtron sequences, we predicted their splicing from the EGFPm coding context. We used the SpliceDB, Softberry program for splicing donor and acceptor site predictions [44,45] and the Human Splice finder for branch point analysis [46]. For structural and delta G (Gibbs free energy) predictions, we used the mFold program [47]. We selected four artificial mirtrons for subsequent investigations, with variable structure and prediction parameters (Supplementary Figures S1–S4). Regarding experimental studies, experiments with three parallels were repeated at least twice. For statistical analysis, a two-sided Student’s t-test was performed.

### 2.2. Plasmid Constructs

For the expression of artificial mirtrons, oligonucleotides corresponding to the sense and antisense sequence of the specific mirtrons were hybridized to form a double-stranded DNA, then inserted as an artificial intron into the PvuII site of EGFPm by blunt-end ligation [39,48]. As a control, we used the third intron of the mouse IgCε gene [49] or a canonical intronic miRNA (mir-33b), as we described earlier [39]. For luciferase constructs, hybridized double-stranded oligonucleotides for the wild type and the seed region mutated target sequences of corresponding mirtrons were ligated between the XhoI/NotI restriction sites of *Renilla* luciferase 3′-UTR in the psiCHECK2 vector (Promega, Madison, WI, USA). For ABCG2 experiments, a previously established pCDNA3.1_ABCG2 plasmid was used [50]. All plasmid constructs were verified by Sanger sequencing.

### 2.3. Cell Cultures and Manipulation

HeLa cell lines were maintained in Dulbecco’s modified Eagle’s medium (DMEM, cat. #31966047) supplemented with 10% of fetal bovine serum (cat. #10500064), 1% of L-glutamine (cat. #25030081), and 1% of penicillin/streptomycin (cat. # 15070063, all from Thermofisher Scientific) using standard cell culture methodology. Cells were transfected with FuGENE^®^ HD reagent (Roche Applied Science, Penzberg, Germany) in a 6-well or 24-well plate, according to the manufacturer’s instruction. To stain the cell nuclei, 10 μM of the Hoechst 33342 dye was used according to the standard protocol. EGFP and Hoechst fluorescence was detected by an IX51 fluorescence microscope (Olympus, Shinjuku City, Tokyo, Japan).

To establish cell lines stably expressing EGFPm-mirtron constructs, we applied the *Sleeping Beauty* transposon-based gene delivery technology as described earlier [51]. Following transfection, cells were sorted for EGFP positivity at day 8 and subsequently at day 15 using a FACS Aria High Speed Cell Sorter (Beckton-Dickinson, Franklin Lake, NJ, USA) to obtain homogenously expressing cell populations. Stable expression was also checked by subsequent FACS analyses. The established cell lines were further used for mRNA level and western blot experiments.

### 2.4. RNA Analysis

Total RNA was isolated from cultured cells using Trizol reagent (Invitrogen, Waltham, MA, USA). To remove genomic DNA contaminations, RNA samples were treated with DNaseI (New England Biolabs, Ipswich, MA, USA) at 37 °C for 1 h. For cDNA preparations, 1 µg of total RNA was reverse transcribed with random primers using High Capacity cDNA Reverse Transcription Kit (Thermofisher Scientific, Waltham, MA, USA). For splicing experiments, a polymerase chain reaction was performed on cDNA prepared from transiently transfected cells (1000 ng of artificial mirtron expressing plasmids were transfected into cells in a 6-well plate), using the following primers: 5′–TTCTTCAAGTCCGCCATGCC (forward) and 5′–ACTTGTACAGCTCGTCCATGCCG (reverse). To carry out real-time quantitative PCR (qPCR), we used specific TaqMan^®^ assays and reagents. Reactions were performed on StepOne™ or StepOnePlus™ platforms, according to the manufacturer’s instructions (Thermofisher Scientific). For relative quantitation, the ∆∆Ct method was applied, and we used the RPLP0 mRNA (catalog number: Hs9999902_m1) as endogenous control. For *Renilla* mRNA level experiments, cells were transfected with 500 ng of respective sensor/mutant sensor expressing plasmid in a 6-well plate. For *Renilla* luciferase mRNA detection, a custom-made TaqMan assay was used, containing the following primers: 5′-CGAGTGGCCTGACATCGA (forward), 5′-ACGAAGAAGTTATTCTCAAGCACCAT (reverse) and 5′-CAGGGCGATATCCTC (probe, with 5′-FAM and 3′-MGB labeled). For firefly luciferase mRNA detection, the following custom-made assay was used: 5′-GCTTCGAGGAGGAGCTGTTC (forward), 5′-CCAGCAGGGCAGACTGAATTT (reverse) and 5′-CAGCCTGCAAGACTAC (probe, with 5′-FAM and 3′-MGB labeled). For ABCG2 mRNA level measurements, 1000 ng of ABCG2 expressing and 500 ng of psiCHECK2 plasmids were co-transfected into cells, seeded in 6-well plates. For qPCR analysis of ABCG2 mRNA, a pre-developed assay was used (catalog number: Hs01053790_m1).

### 2.5. Luciferase Assay

In each experiment, 300 ng of the mirtron/control expressing plasmids were co-transfected with 15 ng of sensor or mutant sensor luciferase plasmids into cells, seeded on a 24-well plate. Sensors containing two copies of the respective target site were cloned downstream of *Renilla* luciferase in the psiCHECK2 vector. Mutant sensors differ in 3 mismatched nucleotides in the predicted miRNA seed region. Luciferase activity was measured at 48 h posttransfection by a 2030 Multilabel Reader luminometer (PerkinElmer, Waltham, MA, USA) using the Dual-Luciferase Reporter Assay System (Promega). Signal specific for firefly luciferase expressed from the same psiCHECK2 plasmid was used to normalize for transfection efficiency. To fully exclude any non-specific effects, luciferase activities of the sensors were also measured in the presence of an unrelated miRNA (hsa-mir-33b) as non-cognate control.

### 2.6. Western Blot (Immunoblot)

Artificial mirtron and control expressing stable HeLa cell lines were transfected with 500 ng ABCG2 expressing plasmid in a 6-well plate. Cells were lysed and collected 48 h after transfection. After briefly sonicated, samples were run on 7.5% acrylamide gel, then electroblotted onto PVDF membrane (BioRad, Hercules, CA, USA). Membranes were blocked by 5% milk/TBS-Tween and incubated with mouse monoclonal BXP-21 antibody (kindly provided by Dr. George Scheffer) overnight at 4 °C for ABCG2 detection. Next, membranes were incubated in HRP-conjugated Anti-Mouse IgG secondary antibody solution (Jackson’s, cat # 715-035-151) for 1 h at room temperature. For signal detection, an ECL reagent (Thermofisher Scientific) was used, and the membranes were exposed to Agfa films. Monoclonal Anti-β-Actin-Peroxidase antibody (Sigma, cat. #A3854) was used for β-actin detection as a control. Experiments were repeated at least three times, and one representative experiment is shown in the figures. Expression levels were quantified by densitometry of the scanned images using the ImageJ software.

## 3. Results

### 3.1. Artificial Mirtron Design

**Figure 1:**
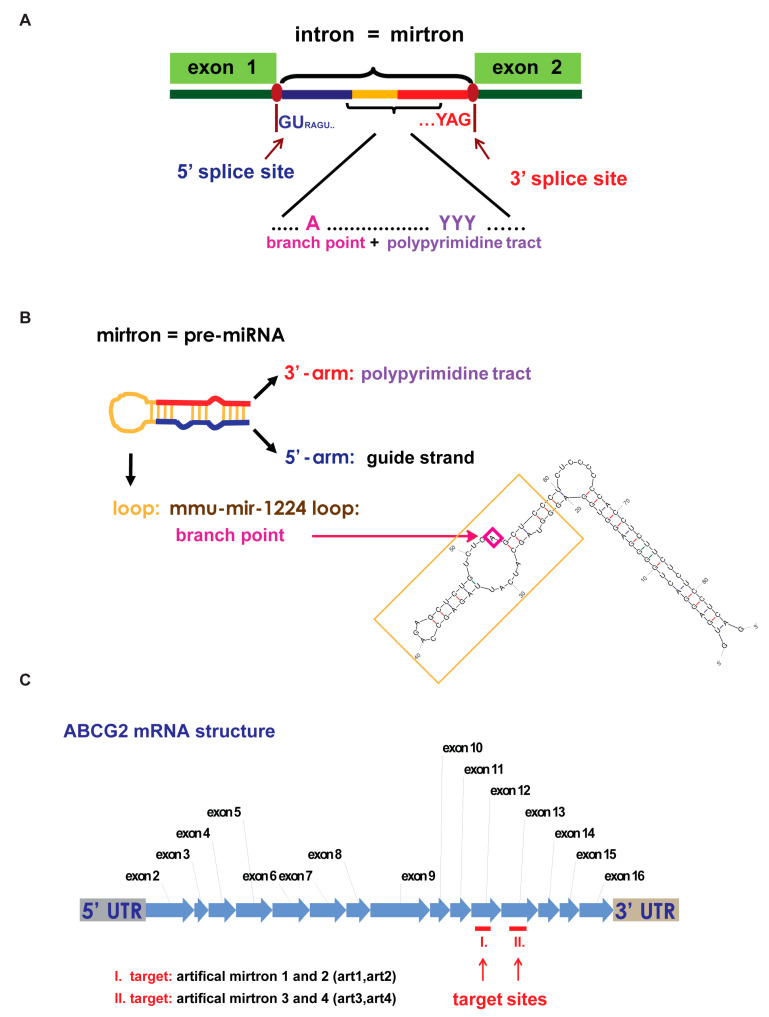
Artificial mirtron design. (**A**) A schematic representation of essential splicing criteria of introns in humans. Since mirtrons are small introns that are liberated by the splicing machinery instead of the Drosha/DGCR8 complex, their sequences should contain the indicated sequence motifs. (**B**) A demonstration of the artificial mirtron design. In the designed mirtronic pre-miRNAs, the 5′-arm gives the potential small guide RNA, and the loop region is originated from *Mus musculus* mir-1224 loop region containing the branch point, while the 3′-arm contains the polypyrimidine tract. The shown putative structure of the mmu-mir-1224 was predicted by the mFold program. (**C**) A schematic representation of the mRNA of ABCG2 membrane transporter protein. Target sites are shown by red, while the experimentally investigated targeting artificial mirtrons (arts) are also indicated.

Although there are numerous advantages of mirtrons as silencers, for the process of artificial mirtron design, some criteria should be considered. First, regarding splicing, a GU 5′-end as 5′ splicing donor site and a (C)AG 3′- end as 3′ acceptor site is advantageous. Then, a functional polypyrimidine tract and a branch point should be placed somewhere in the mirtron sequence (Figure 1A). Additionally, as was mentioned above, there are some features to be considered for proper processing by Dicer and for the loading of the appropriate strand of the small RNA duplex into a functional RISC. Besides, there are some other concerns regarding efficient silencing, such as the complementarity of the small RNA to its target.

Theoretically, the guide strand can be placed either in the 5′- or in the 3′-arm of a mirtron. However, in all cases, we chose the 5′-arm because, in this case, the most important part of the potential guide RNA, the 5′-end and therefore the seed region is well defined by splicing, avoiding potential heterogeneous ends, resulted by Dicer processing. Hence, it is easier to plan target specificity and influence strand selection. Concerning the branch point, we analyzed several mammalian mirtrons and found that some of them have their potential branch point in the loop region, while some have it in the 3′-arm region. We decided to position it in the loop region while the polypyrimidine tract was placed in the 3′-arm. We used the mmu-mir-1224 mirtron loop sequence as the loop of our artificial mirtrons since it had the best scores for branch point and splicing prediction analysis during the design process (Figure 1B). Regarding target site selection, we selected target sequences from the coding region of ABCG2 (Figure 1C) since it was previously shown to be applicable [40] and because our earlier effort to target 3′ UTR did not result in efficient silencing (data not shown). During the design process, we selected AC dinucleotides in the ABCG2 cDNA beside pyrimidine-rich sequences in the 5′ neighborhood to be the potential target of a 5′-arm derived mirtronic small RNA. Thus, the 5′-arm of the artificial mirtron (artmir) is complementary to the target site, the loop region contains the branch point, and the 3′-arm has the polypyrimidine tract (Figure 1B). We designed several sequence variants to test complementarity/silencing ability correlations and chose candidates for experimental investigations by bioinformatic analysis. Here we show four artificial mirtron variants targeting two constitutive exons as potential target sites: art1 and art2 for target I (residing in exon 12), and art3 and art4 for target II (residing in exon 13; Figure 1C).

### 3.2. Investigating Splicing Ability of the Selected Artificial Mirtrons

**Figure 2:**
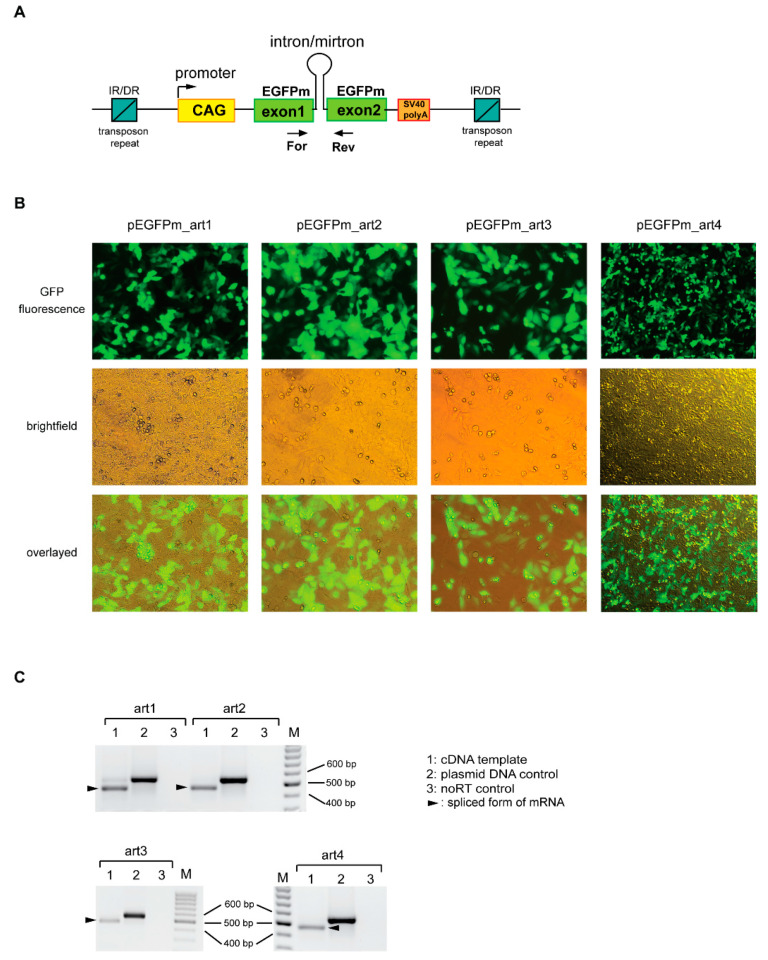
The splicing ability of selected artificial mirtrons (art1–4). (**A**) The *Sleeping Beauty* transposon constructs used for the expression of artificial mirtrons as EGFPm introns. Arrows indicate primers used for RT-PCR. IR/DR: inverted repeat/direct repeat sequences of the transposon. (**B**) Representative fluorescence microscopy images of HeLa cells expressing artmirs. EGFP positive cells indicate successful splicing. (**C**) RT-PCR results for the analysis of splicing. The spliced forms of RNAs are shown by black arrowheads. PCR products from plasmid DNA indicate the size of the unspliced mRNAs. M: DNA ladder as a marker.

For the expression of artificial mirtrons, we used our earlier established expression system [39]. A modified EGFP sequence (EGFPm) was used, of which the coding region was separated into two exons. The artificial mirtrons were cloned as introns between the two exons. Therefore, EGFP fluorescence indicates accurate splicing, and artificial mirtron expression can be easily monitored (Figure 2A).

In the case of all four artmirs, we observed quite strong EGFP expression in the transfected cells, suggesting proper splicing (Figure 2B and Supplementary Figure S5). Investigation of splicing by RT-PCR indeed revealed successful, very efficient splicing. We detected a small amount of unspliced mRNA form only in the case of art1 (Figure 2C). Splicing accuracy was confirmed by sequencing of the gel-purified PCR products (Supplementary Figure S6). Our experimental results were consistent with our splicing predictions of the design phase. We chose sequences with very high values of splicing donor, acceptor and branch point predictions, and among them, art1 had the lowest values (see Supplementary Figures S1–S4).

Since the expression cassette in our plasmid was located inside a *Sleeping Beauty* transposon (Figure 2A), co-transfection with a transposase expressing plasmid allowed us to make stable cell lines by sorting the cells based on the EGFP signal. We successfully established all four artificial mirtron-expressing stable cell lines for further experiments.

### 3.3. Functional Testing of Artificial Mirtrons by Luciferase Reporter Assay

**Figure 3:**
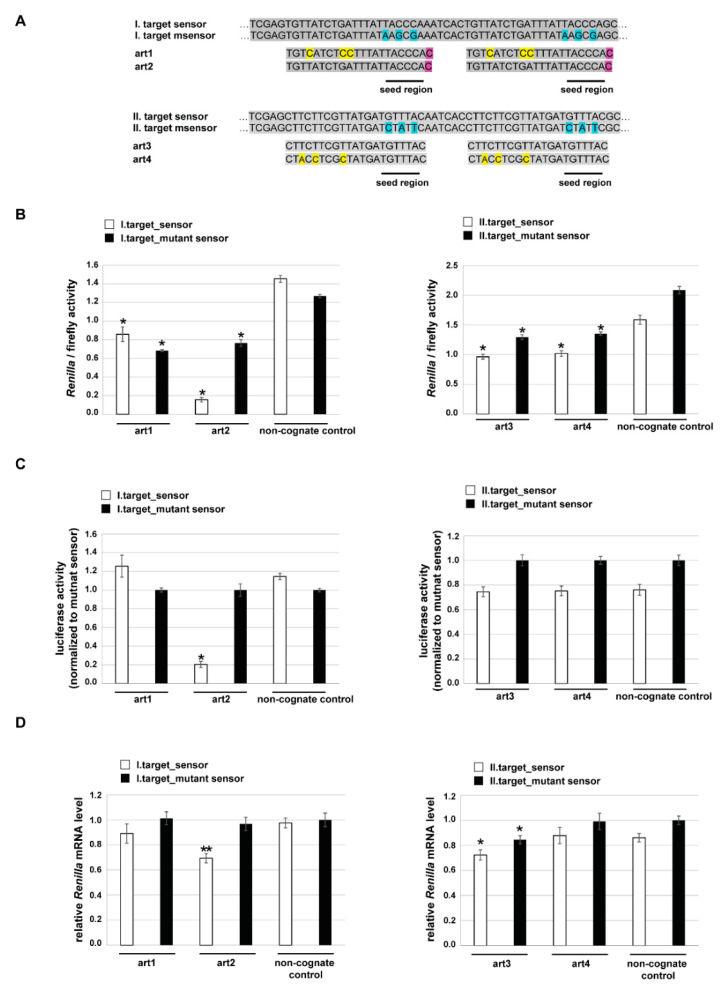
Testing the silencing ability of artificial mirtrons by luciferase assay. (**A**) DNA sequence alignment of luciferase targets and their respective artmirs (sense DNA sequences are shown). The psiCHECK2 vector-based sensor constructs contained two antisense copies of the particular target, cloned downstream of *Renilla* luciferase reporter. Sensors and mutant sensors (msensor) differ in 3 nucleotides in the potential seed region, indicated by the blue background. Art1 and art2 have a mismatch at their first position to the target since an extra G (its complementary ‘C’ in DNA is indicated by purple background) was added to their 5′-end to fulfill mirtron criteria. Art3 is fully complementary to its sensor. Art1 and art4 have extra mismatches in their middle and 3′-end regions (indicated by yellow background), compared to their counterparts, art2 and art3, respectively. (**B**) Dual-luciferase assay measurements to assess the silencing ability of the designed artmirs. Mean values of at least 3 parallel experiments are shown, and error bars represent standard deviations; *: *p* < 0.001 relative to respective control. (**C**) To examine ‘seed-specific’ silencing, luciferase activity (*Renilla*/firefly) of sensor-containing experiments are normalized to the respective mutant sensor values (set to 1 for each mirtron). *: *p* < 0.001, relative to respective normalizing control. (**D**) Luciferase mRNA level measurements by qPCR. Error bars represent standard deviations; *: *p* < 0.05, **: *p* < 0.01, relative to respective control.

Since all of the examined artmirs could be effectively spliced out from the host gene, we tested their ability to silence gene expression. We used luciferase sensor assays, for which two copies of the particular target were cloned downstream of the *Renilla* luciferase coding region. Besides the fully complementary seed region containing sensor, we also used a mutant sensor bearing 3 mismatches in the seed region to check seed region specificity (Figure 3A).

For target I., we detected downregulation of both sensor types in the case of both artmirs compared to a non-cognate control (Figure 3B left). Mutant sensors were silenced at a similar extent, by ~30%. Regarding the sensor (having a fully complementary seed region), both artmirs could achieve repression, but art2 had a much higher silencing capacity on it. The extent of the repression was ~43% for art1, whereas ~87% for art2. However, if we compare the downregulation of the sensor to the mutant sensor, we see a significant difference only in the case of art2 (Figure 3C left). The knockdown efficiency, in this case, was also high, more than 80%. For target II., we detected downregulation of both sensor types in the case of the corresponding artmirs compared to a non-cognate control (Figure 3B right). Silencing efficiencies were similar (~30%) among art3 and art4 in the case of both sensor types. However, a comparison of the sensor repression to the mutant sensor repression indicated no differences between artmirs and the non-cognate control (Figure 3C right).

As mentioned above, we designed artmirs to have different sequence complementarity to the target (Figure 3A), and we wanted to test whether there is a difference in their mechanism of silencing: is the observed luciferase repression realized by the cleavage/destabilization of the target mRNA or via translational repression? For this, we measured the mRNA level of *Renilla* luciferase by quantitative PCR. In the case of target I., no significant decrease could be detected in the mutant sensor containing *Renilla* luciferase mRNA. Regarding the sensor containing *Renilla* luciferase mRNA, we detected a significant decrease only in the case of art2, where a ~30% reduction was observed, compared to the control (Figure 3D left). Concerning target II., there was no change in either sensor or mutant sensor containing *Renilla* luciferase mRNA level when art4 was expressed. However, in the case of art3, we observed a slight, ~15% reduction of both sensor types (Figure 3D right).

### 3.4. Targeting ABCG2 Expression by Artificial Mirtrons

**Figure 4:**
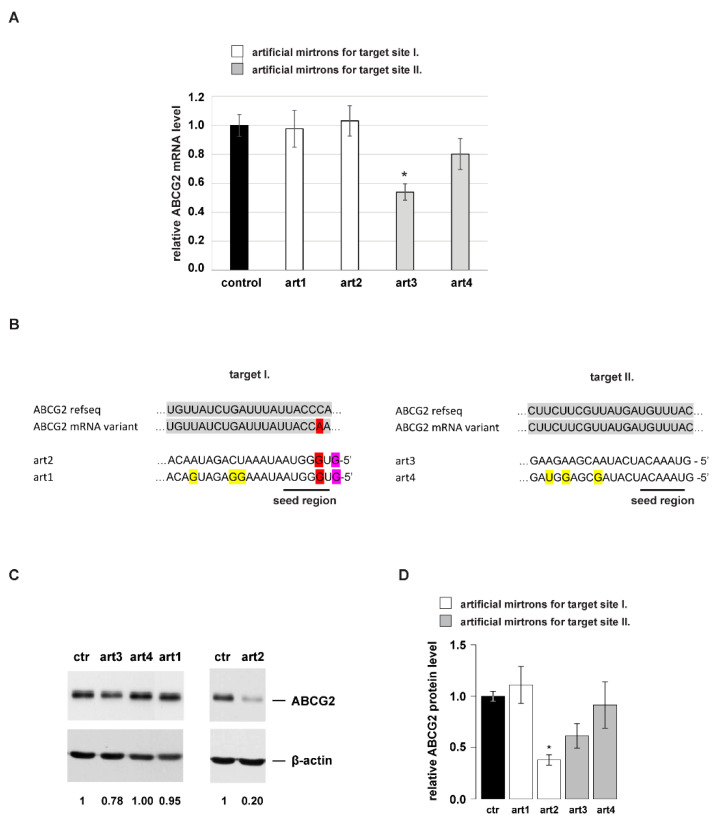
Targeting ABCG2 expression by artificial mirtrons. (**A**) Investigating the mirtron-induced silencing of ABCG2 mRNA by qPCR. Mean values are shown, and error bars represent standard deviations; *: *p* < 0.005 relative to respective control. (**B**) An illustration of artmirs complementary to the respective ABCG2 mRNA target site. Artmirs were designed based on the reference sequence (NM_004827.3); however, experiments were carried out with a common ABCG2 variant, therefore having a mismatch in target I. sequence (indicated by red background). Purple and yellow backgrounds indicate nucleotides, as in Figure 3A. (**C**) Examination of ABCG2 protein repression by artmirs, a representative western blot experiment is shown; normalized values of ABCG2 protein levels are shown under the gel image. (**D**) Relative protein levels measured after ABCG2 silencing by the artificial mirtron constructs. The mean values of 3 independent experiments are shown for artmirs, and the mean value of 5 independent experiments is shown for control (ctr). Error bars represent S.E.M., *: *p* < 0.005.

Next, we investigated the ability of the designed mirtrons to silence the expression of the human ABCG2 gene. First, we examined their impact on ABCG2 mRNA expression. Compared to the control, we observed a significant decrease, ~45% only in the case of art3 (Figure 4A).

Based on our previous luciferase experiments, we expected a reduction in the mRNA level also for art2 (see Figure 3D). However, the human ABCG2 gene has various polymorphisms compared to the reference sequence and sequencing the target site I. of our expression construct revealed the presence of one silent polymorphism (CCC > CCA, Pro480Pro). This extra mismatch in the 3rd nucleotide position of the seed region of art1 and art2 compared to the ABCG2 mRNA could well explain the results (Figure 4B).

Finally, we tested if the designed artmirs can influence ABCG2 expression at the protein level. For this, we carried out western blot experiments. We could detect a significant reduction in ABCG2 protein expression by art2 and a much less prominent decrease by art3. However, in the case of art1 and art4, carrying extra mismatches in the 3′ and the middle region of the miRNAs, we observed no significant changes compared to the control (Figure 4C,D).

## 4. Discussion

In this study, we aimed to design artificial mirtrons to silence ABCG2 expression and investigate some sequential features that could influence efficient silencing. As was mentioned above, mirtrons can serve as useful tools for gene silencing, and they can be exploited in particular when genome editing is not amenable or silencing should be reversible. As artificial introns, they could be placed in various reporter genes, or for therapeutic applications, they may be combined with other genes of interest, achieving more than one genetic effect simultaneously with one expression cassette. Here, we present an artificial mirtron-based approach by which a significant silencing effect can be achieved on the ABCG2 multidrug transporter protein using the EGFP host protein. By developing and combining with appropriate Pol II promoters, it can serve as a useful tool for the investigation of ABCG2 function in various stem cells, including human embryonic stem cells and cells exhibiting the so-called “side-population phenotype” [6,8,52]. To date, there are only a few studies addressing the development of artificial conventional mirtrons to silence gene expression and their potential use in therapeutic applications. In those studies, the silencing effect of artmirs was investigated mostly in luciferase reporter assays and at the target mRNA level [40,41,43]. In a subsequent article, the potential application of 3′-tailed artificial mirtrons was studied, where in addition to the mRNA level, an efficient decrease could also be detected on the indirectly measured protein level of VEGFA [42]. Our data further strengthen the applicability of artificial mirtrons as gene silencers since our careful design could result in mirtrons efficiently reducing the ABCG2 expression when the target protein level was measured directly.

When examining the designed artmirs, interesting sequential features could be observed. We designed artmirs complementary to their targets or having mismatches at various positions. Using mutant sensors in luciferase assays revealed that 3 nucleotide mismatches in the seed region did not abolish silencing at the protein level since all four artmirs could have a silencing effect on the target, compared to the non-cognate control (Figure 3B). The extent of silencing was comparable to that measured on the sensor for art1, art3 and art4, indicating ‘non-seed-specific’ repression. However, in the case of art2, a strong ‘seed-specific’ silencing effect was observed (~80% reduction). Regarding mRNA levels, we detected a significant reduction in the *Renilla* mRNA level only in the case of art2 and art3. Art2 reduced its sensor mRNA level by ~30%, while art3 had a smaller effect but surprisingly on both sensor types (Figure 3D). Worth noting, that while art3 is fully complementary to its target, art2 has one mismatch outside the seed region, at the first position, due to the mirtron design rule (having G at the 5′-end). However, despite this mismatch, art2 decreased its sensor mRNA level and more extensively than art3. Nevertheless, when the target is located in the original genomic context, the ability of art2 to reduce the ABCG2 mRNA level was abolished by an additional mismatch positioned in the seed region (3rd position, Figure 4B). In summary, data of the luciferase experiments suggest that art1 and art4 silenced their targets via translational repression, while art2 and art3 could accelerate the degradation of their target mRNA to some extent, even if having mismatches to the target (1st nucleotide of art2 in its sensor, or seed mismatches in mutant sensor of art3).

Concerning ABCG2, only art3 repressed its mRNA level (~45%), but it only resulted in a slight reduction of the amount of protein. Conversely, art2 had no impact on mRNA level but exhibited a quite strong repression at the protein level. In contrast to these, art1 and art4 had no effect on either ABCG2 mRNA or protein level. Considering the sequence environment, ABCG2 mRNA has one, while *Renilla* mRNA has two target sites; however, art3 can regulate the former one more efficiently (~45% versus 15%). Nevertheless, it is worth noting, that in ABCG2 mRNA, the target sequence resides in the cDNA region instead of the 3′ UTR. The results indicate that flanking sequences could strongly influence the miRNA effect. In the natural mRNA context, art3 achieves a slight decrease in ABCG2 protein level, most probably by degrading its mRNA, while art2 operates only by translational repression.

Regarding our data, we noticed that in the case of the ‘miRNA-mimic’ artmirs (art1 and art4) containing mismatches in the 3′ and the middle region, losing base pairing at the investigated positions did not accelerate silencing either at luciferase or at the ABCG2 protein level, compared to their respective counterparts (art2 and art3). In addition, we also noticed that the presence of rare polymorphisms in the target region should also be considered since they could influence base pairing and thereby efficient silencing of the designed mirtrons (Figure 4B). On the other hand, some other sequential features can be very useful during artificial mirtron design: for example, our data support the possibility of adding a non-complementary G to the 5′-end of a mirtron without decreasing silencing ability, which is very important and useful since it is a strong mirtron criterion [35,36].

Taken together, some of our results using artificial mirtrons are in line with earlier data, such as the reduction of the target mRNA level in the case of full complementarity between the target and the small RNA [29,53]. However, we observed some additional, not expected features, e.g., a reduction in the target mRNA level when the first nucleotide of the small RNA is not complementary. Further experiments are needed to reveal whether these findings are common phenomena or a consequence of the given target sequence and/or its context, which may have different accessibility by the RISC. Another explanation could be an altered RISC assembly when the various small RNA guides are preferentially associated with different Argonaute proteins. Notably, the most successful silencer artmir of LRRK2 was associated with the greatest amount to AGO4 [41].

## 5. Conclusions

In summary, using our artificial mirtron design and testing scheme, we could successfully establish an efficient silencing system for the ABCG2 multidrug transporter. In addition, we observed important new sequential-functional features of the designed mirtrons. Our silencing system could be directly applied to study the function of this membrane protein in several in vitro or in vivo models. Moreover, combining the artmirs with host proteins other than EGFP, this system would also be suitable for versatile, functional studies in stem cells, where ABCG2 plays an important yet not fully understood role. However, apart from the concrete established model system, we believe that our mirtron design pipeline could also be efficiently applied to target other genes in future studies.

## Data Availability

All data are included in the manuscript or in the supplementary materials.

## Supplementary Materials

The following are available online at https://www.mdpi.com/article/10.3390/genes12071068/s1, Figure S1: Predicted splicing parameters and structure of artificial mirtron 1 (art1), Figure S2: Predicted splicing parameters and structure of artificial mirtron 2 (art2), Figure S3: Predicted splicing parameters and structure of artificial mirtron 3 (art3), Figure S4: Predicted splicing parameters and structure of artificial mirtron 4 (art4), Figure S5. Microscopy images of untransfected HeLa cells, Figure S6: Sequence alignment of RT-PCR products of spliced mRNAs versus their unspliced form.
