# Supplementary material for: Posttranscriptional Regulation of the Human ABCG2 Multidrug Transporter Protein by Artificial Mirtrons
Source: Genes (Basel). 2021 Jul 13;12(7):1068. doi: 10.3390/genes12071068 (PMC8307164; doi:10.3390/genes12071068)

A

>EGFPm_art1
ATGGTGAGCAAGGGCGAGGAGCTGTTCACCGGGGTGGTGCCCATCCTGGTCGAG
CTGGACGGCGACGTAAACGGCCACAAGTTCAGCGTGTCCGGCGAGGGCGAGGGC
GATGCCACCTACGGCAAGCTGACCCTGAAGTTCATCTGCACCACCGGCAAGCTGC
CCGTGCCCTGGCCCACCCTCGTGACCACCCTGACCTACGGCGTGCAGTGCTTCAG
CCGCTACCCCGACCACATGAAGCAGCACGACTTCTTCAAGTCCGCCATGCCCGAA
GGCTACGTCCAGGAGCGCACCATCTTCTTCAAGGACGACGGCAACTACAAGACCC
GcGCCGAGGTGAAGTTCGAGGGCGACACCCTGGTGAACCGCATCGAGCTGAAGG
GCATCGACTTCAAGGAGGACGGCAACATCCTGGGGCACAAGCTGGAGTACAACT
ACAACAGCCACAACGTCTATATCATGGCCGACAAGCAGAAGAACGGCATCAAGG
TGAACTTCAAGATCCGCCACAACATCGAGGACGGCAGCGTGCAGGTGGGTAATA
AAGGAGATGACAGGTAGCATCATTAGAGCCAGAGCTCTGTCTCAGCTCCTCTTAT
CTCTTTTATTACCCAGCTGGCCGACCACTACCAGCAGAACACCCCCATCGGCGAC
GGCCCCGTGCTGCTGCCCGACAACCACTACCTGAGCACCCAGTCCGCCCTGAGCA
AAGACCCCAACGAGAAGCGCGATCACATGGTCCTGCTGGAGTTCGTGACCGCCG
CCGGGATCACTCTCGGCATGGACGAGCTGTACAAGTAA

B

```
Acceptor(AG) sites. Treshold      0.000 (100%).
       1 P:      210 W:  4.10 Seq: tacggcgtgcAGtgcttcagcc
       2 P:      306 W:  0.23 Seq: atcttcttcaAGgacgacggca
       3 P:      615 W: 10.47 Seq: tttattacccAGctggccgacc
 Donor(GT) sites. Treshold      0.079 (100%).
       1 P:        4 W: 10.02 Seq: -------atgGTgagcaagggc
       2 P:       67 W:  3.30 Seq: ggacggcgacGTaaacggccac
       3 P:      337 W:  5.96 Seq: ccgcgccgagGTgaagttcgag
       4 P:      361 W:  1.90 Seq: cgacaccctgGTgaaccgcatc
       5 P:      490 W:  6.94 Seq: cggcatcaagGTgaacttcaag
       6 P:      535 W: 11.28 Seq: cagcgtgcagGTgggtaataaa
       7 P:      539 W:  1.34 Seq: gtgcaggtggGTaataaaggag
       8 P:      558 W:  8.20 Seq: gagatgacagGTagcatcatta
       9 P:      798 W:  4.10 Seq: agctgtacaaGTaa--------
```

C

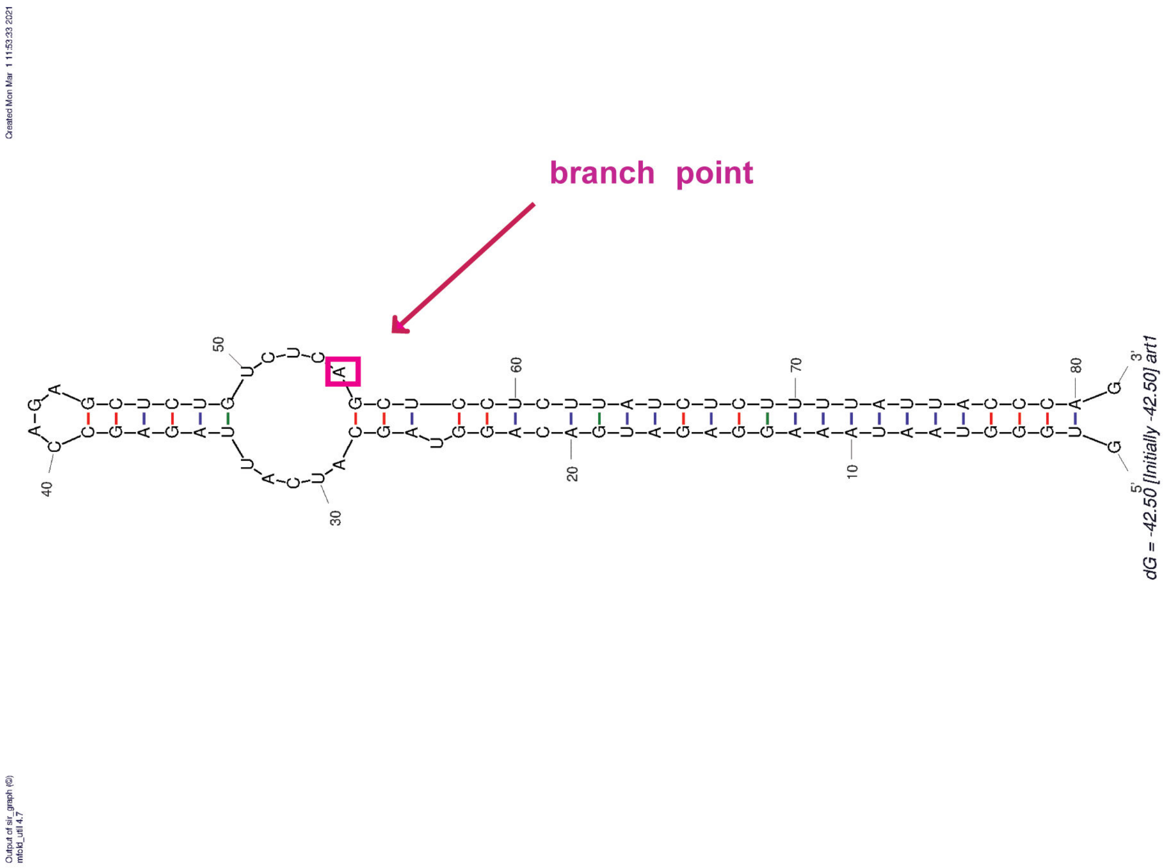

Supplement: Supplementary file 1 [file genes-12-01068-s001.zip › Figure S1.pdf]
