# Supplementary material for: Posttranscriptional Regulation of the Human ABCG2 Multidrug Transporter Protein by Artificial Mirtrons
Source: Genes (Basel). 2021 Jul 13;12(7):1068. doi: 10.3390/genes12071068 (PMC8307164; doi:10.3390/genes12071068)

**A**

>EGFPm_art2
ATGGTGAGCAAGGGCGAGGAGCTGTTCACCGGGGTGGTGCCCATCCTGGTCGAG
CTGGACGGCGACGTAAACGGCCACAAGTTCAGCGTGTCCGGCGAGGGCGAGGGC
GATGCCACCTACGGCAAGCTGACCCTGAAGTTCATCTGCACCACCGGCAAGCTGC
CCGTGCCCTGGCCCACCCTCGTGACCACCCTGACCTACGGCGTGCAGTGCTTCAG
CCGCTACCCCGACCACATGAAGCAGCACGACTTCTTCAAGTCCGCCATGCCCGAA
GGCTACGTCCAGGAGCGCACCATCTTCTTCAAGGACGACGGCAACTACAAGACCC
GcGCCGAGGTGAAGTTCGAGGGCGACACCCTGGTGAACCGCATCGAGCTGAAGG
GCATCGACTTCAAGGAGGACGGCAACATCCTGGGGCACAAGCTGGAGTACAACT
ACAACAGCCACAACGTCTATATCATGGCCGACAAGCAGAAGAACGGCATCAAGG
TGAACTTCAAGATCCGCCACAACATCGAGGACGGCAGCGTGCAGGTGGGTAATA
AATCAGATAACAGGTAGCATCATTAGAGCCAGAGCTCTGTCTCAGCTCCTGTTAT
CTTCTTTATTCCCCAGCTGGCCGACCACTACCAGCAGAACACCCCCATCGGCGAC
GGCCCCGTGCTGCTGCCCGACAACCACTACCTGAGCACCCAGTCCGCCCTGAGCA
AAGACCCCAACGAGAAGCGCGATCACATGGTCCTGCTGGAGTTCGTGACCGCCG
CCGGGATCACTCTCGGCATGGACGAGCTGTACAAGTAA

**B**

```
Acceptor(AG) sites. Treshold      0.000 (100%).
      1 P:     210 W:  4.10 Seq: tacggcgtgcAGtgcttcagcc
      2 P:     306 W:  0.23 Seq: atcttcttcaAGgacgacggca
      3 P:     615 W: 12.60 Seq: tttattccccAGctggccgacc
Donor(GT) sites. Treshold      0.079 (100%).
      1 P:       4 W: 10.02 Seq: -------atgGTgagcaagggc
      2 P:      67 W:  3.30 Seq: ggacggcgacGTaaacggccac
      3 P:     337 W:  5.96 Seq: ccgcgccgagGTgaagttcgag
      4 P:     361 W:  1.90 Seq: cgacaccctgGTgaaccgcatc
      5 P:     490 W:  6.94 Seq: cggcatcaagGTgaacttcaag
      6 P:     535 W: 11.28 Seq: cagcgtgcagGTgggtaataaa
      7 P:     539 W:  1.34 Seq: gtgcaggtggGTaataaatcag
      8 P:     558 W:  8.20 Seq: cagataacagGTagcatcatta
      9 P:     798 W:  4.10 Seq: agctgtacaaGTaa--------
```

**C**

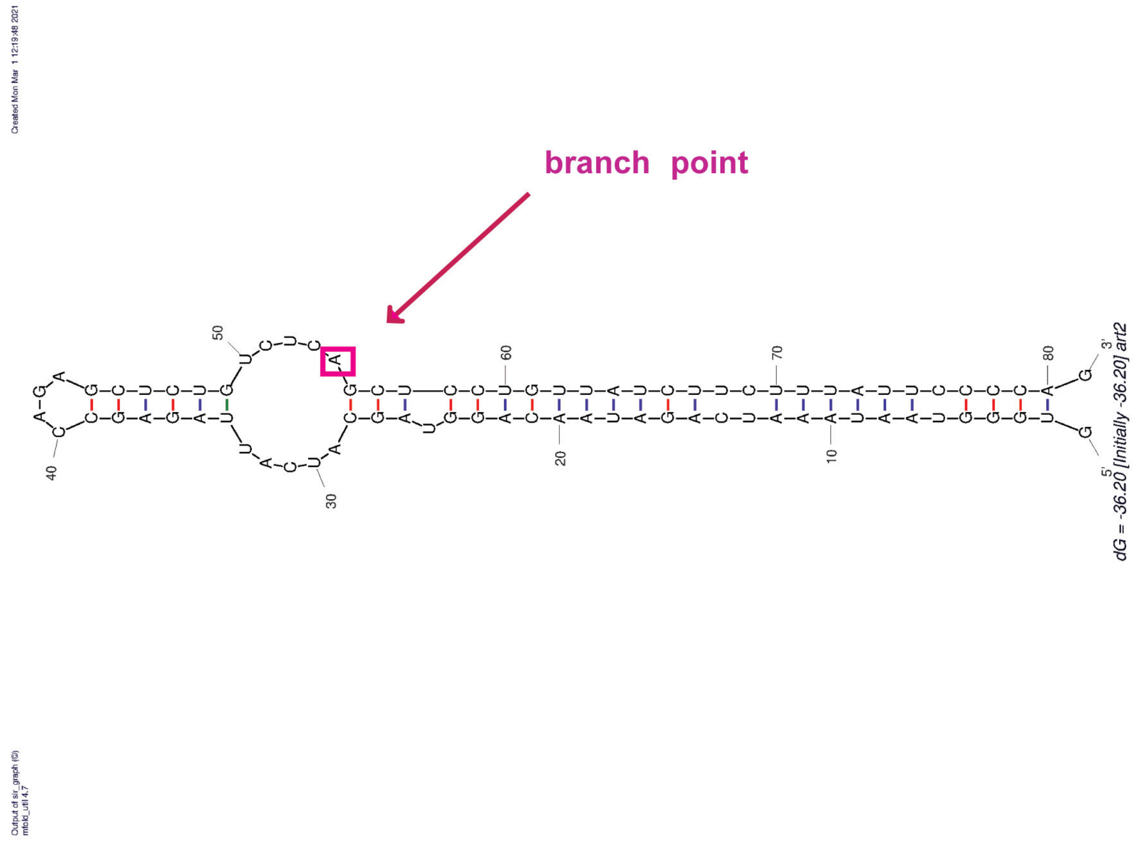

Supplement: Supplementary file 1 [file genes-12-01068-s001.zip › Figure S2.pdf]
