# Supplementary material for: Posttranscriptional Regulation of the Human ABCG2 Multidrug Transporter Protein by Artificial Mirtrons
Source: Genes (Basel). 2021 Jul 13;12(7):1068. doi: 10.3390/genes12071068 (PMC8307164; doi:10.3390/genes12071068)

A

>EGFPm_art3
ATGGTGAGCAAGGGCGAGGAGCTGTTCACCGGGGTGGTGCCCATCCTGGTCGAG
CTGGACGGCGACGTAAACGGCCACAAGTTCAGCGTGTCCGGCGAGGGCGAGGGC
GATGCCACCTACGGCAAGCTGACCCTGAAGTTCATCTGCACCACCGGCAAGCTGC
CCGTGCCCTGGCCCACCCTCGTGACCACCCTGACCTACGGCGTGCAGTGCTTCAG
CCGCTACCCCGACCACATGAAGCAGCACGACTTCTTCAAGTCCGCCATGCCCGAA
GGCTACGTCCAGGAGCGCACCATCTTCTTCAAGGACGACGGCAACTACAAGACCC
GcGCCGAGGTGAAGTTCGAGGGCGACACCCTGGTGAACCGCATCGAGCTGAAGG
GCATCGACTTCAAGGAGGACGGCAACATCCTGGGGCACAAGCTGGAGTACAACT
ACAACAGCCACAACGTCTATATCATGGCCGACAAGCAGAAGAACGGCATCAAGG
TGAACTTCAAGATCCGCCACAACATCGAGGACGGCAGCGTGCAGGTAAACATCA
TAACGAAGAAGGGTAGCATCATTAGAGCCAGAGCTCTGTCTCAGCTCCCTTCTTC
CTTATCATCTTTCAGCTGGCCGACCACTACCAGCAGAACACCCCCATCGGCGACG
GCCCCGTGCTGCTGCCCGACAACCACTACCTGAGCACCCAGTCCGCCCTGAGCAA
AGACCCCAACGAGAAGCGCGATCACATGGTCCTGCTGGAGTTCGTGACCGCCGCC
GGGATCACTCTCGGCATGGACGAGCTGTACAAGTAA

B

```
Acceptor(AG) sites. Treshold      0.000 (100%).
       1 P:      210 W:  4.10 Seq: tacggcgtgcAGtgcttcagcc
       2 P:      306 W:  0.23 Seq: atcttcttcaAGgacgacggca
       3 P:      614 W: 11.43 Seq: atcatctttcAGctggccgacc
       4 P:      635 W:  0.10 Seq: cactaccagcAGaacaccccca
 Donor(GT) sites. Treshold      0.079 (100%).
       1 P:        4 W: 10.02 Seq: -------atgGTgagcaagggc
       2 P:       67 W:  3.30 Seq: ggacggcgacGTaaacggccac
       3 P:      337 W:  5.96 Seq: ccgcgccgagGTgaagttcgag
       4 P:      361 W:  1.90 Seq: cgacaccctgGTgaaccgcatc
       5 P:      490 W:  6.94 Seq: cggcatcaagGTgaacttcaag
       6 P:      535 W: 12.40 Seq: cagcgtgcagGTaaacatcata
       7 P:      557 W:  1.62 Seq: acgaagaaggGTagcatcatta
       8 P:      797 W:  4.10 Seq: agctgtacaaGTaa--------
```

C

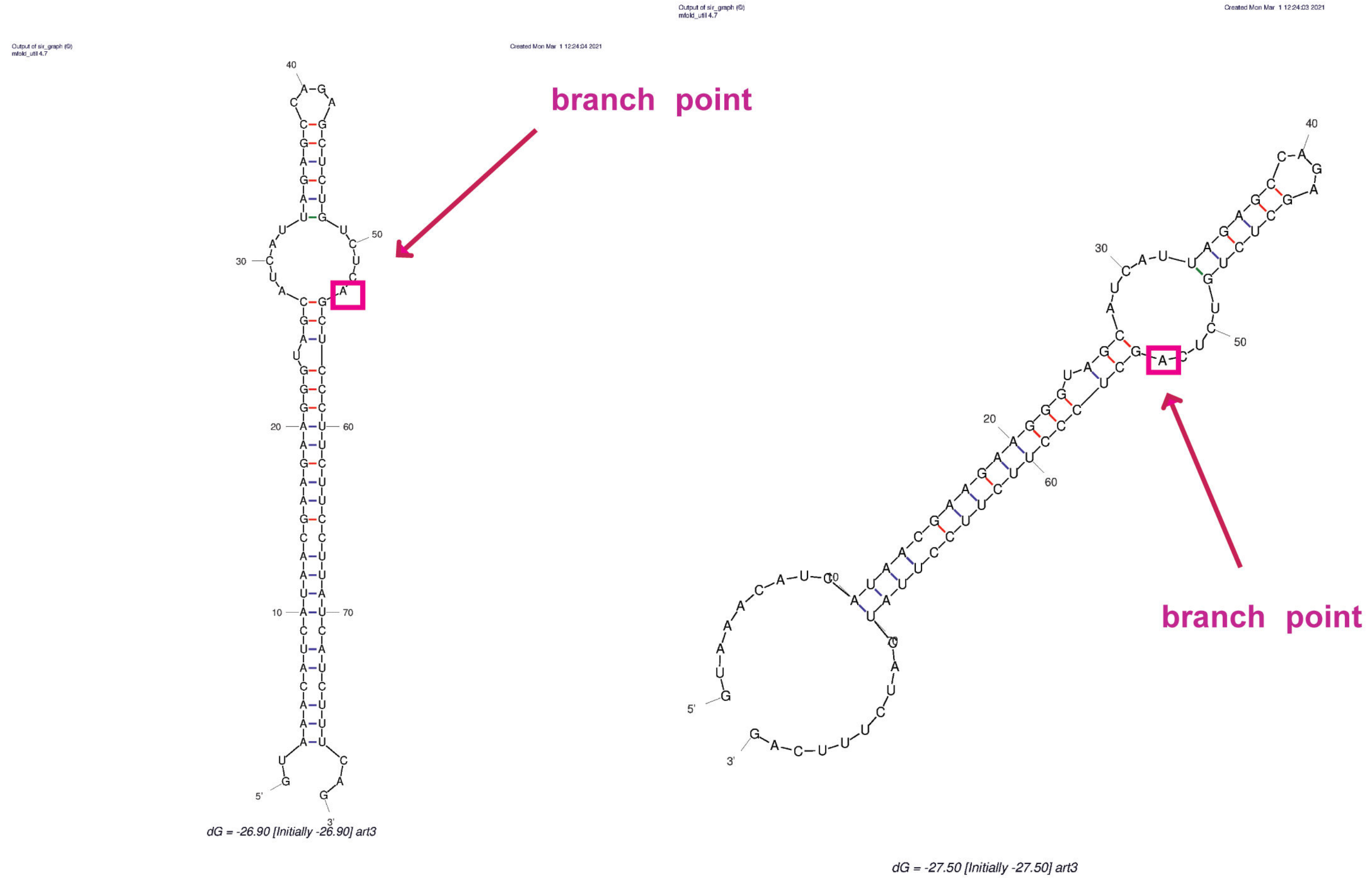

Supplement: Supplementary file 1 [file genes-12-01068-s001.zip › Figure S3.pdf]
