# Supplementary material for: Posttranscriptional Regulation of the Human ABCG2 Multidrug Transporter Protein by Artificial Mirtrons
Source: Genes (Basel). 2021 Jul 13;12(7):1068. doi: 10.3390/genes12071068 (PMC8307164; doi:10.3390/genes12071068)

Untransfected HeLa cells

20x magnification **40x magnification**

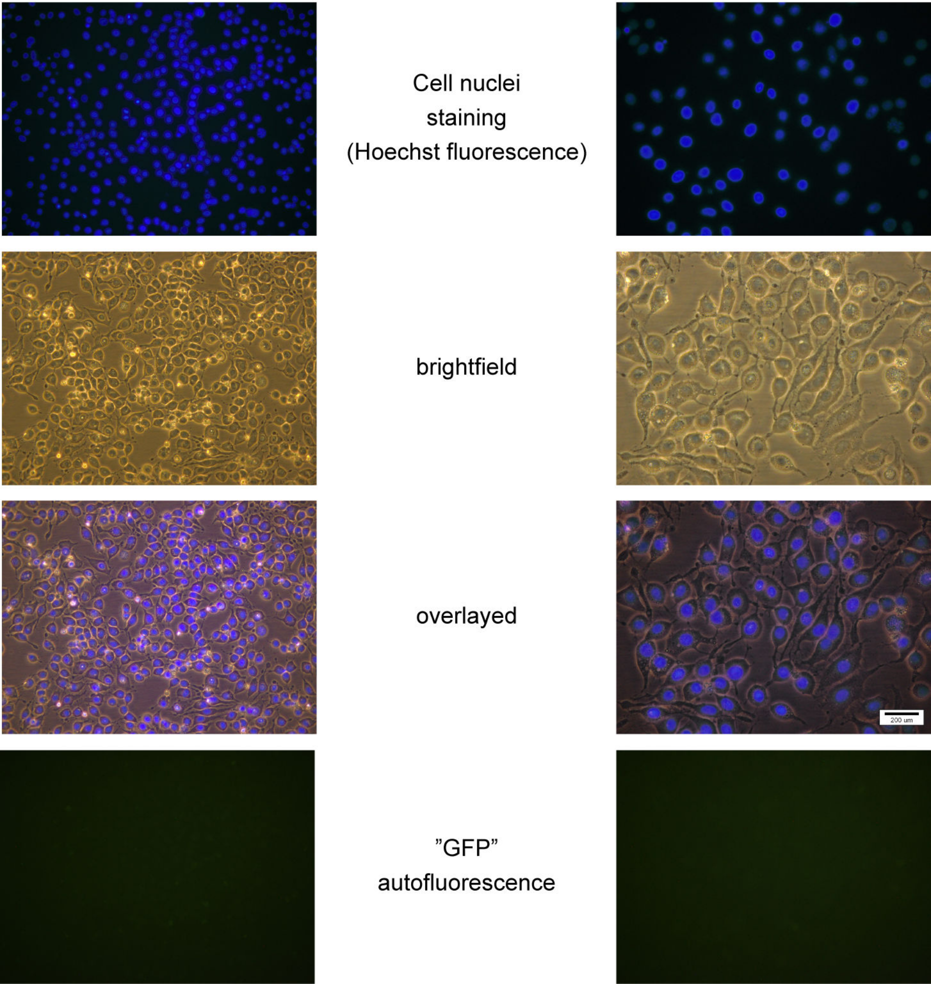

Supplement: Supplementary file 1 [file genes-12-01068-s001.zip › Figure S5.pdf]
