# Supplementary material for: Posttranscriptional Regulation of the Human ABCG2 Multidrug Transporter Protein by Artificial Mirtrons
Source: Genes (Basel). 2021 Jul 13;12(7):1068. doi: 10.3390/genes12071068 (PMC8307164; doi:10.3390/genes12071068)
Supplement: Supplementary file 1 [file genes-12-01068-s001.zip › Figure S6.pdf]

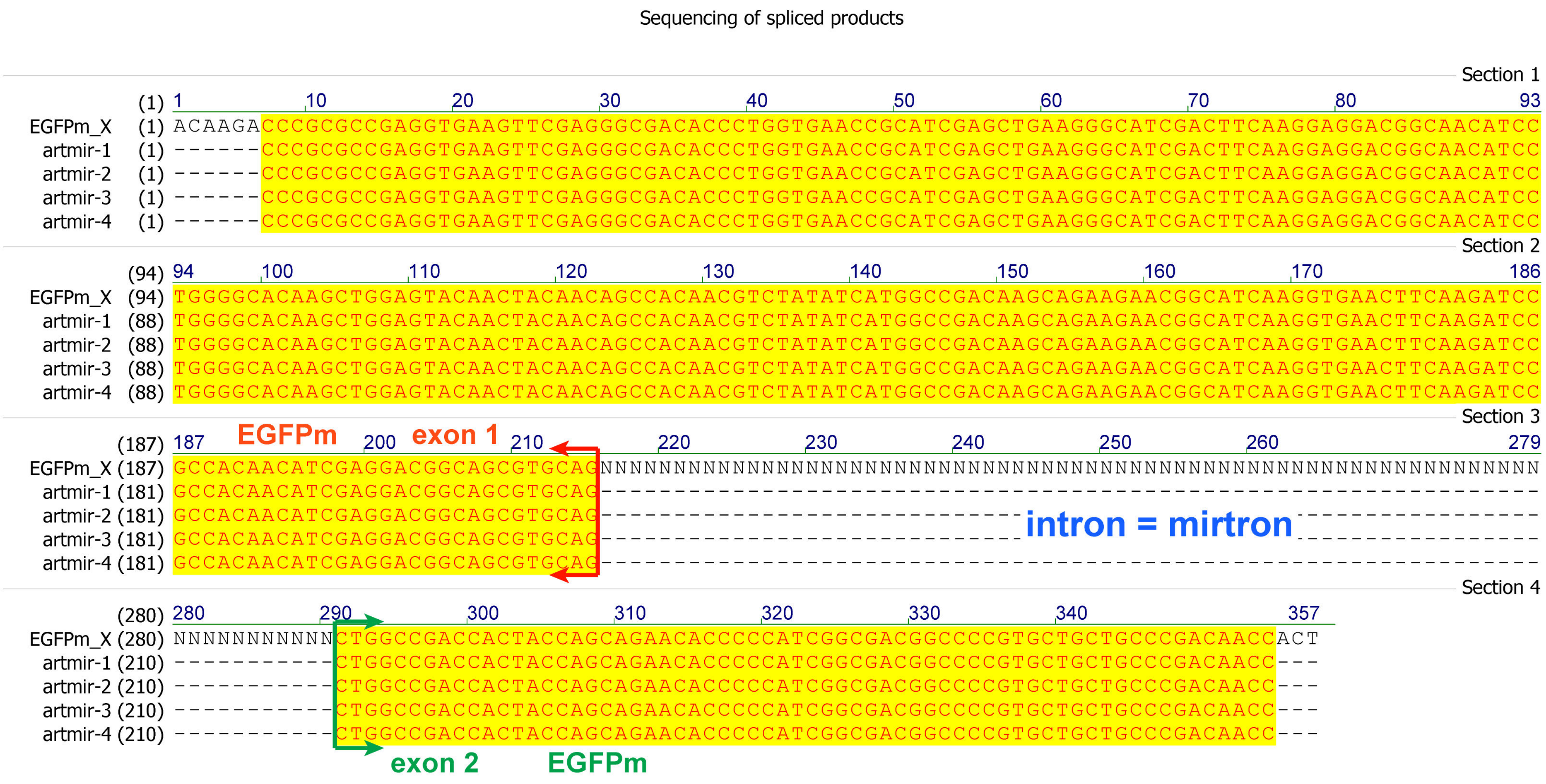
Sequencing of spliced products
Section 1
(1) 1 10 20 30 40 50 60 70 80 93
EGFPm_X (1) ACAAGACCCGCGCCGAGGTGAAGTTCGAGGGCGACACCCTGGTGAACCGCATCGAGCTGAAGGGCATCGACTTCAAGGAGGACGGCAACATCC
artmir-1 (1) ------CCCGCGCCGAGGTGAAGTTCGAGGGCGACACCCTGGTGAACCGCATCGAGCTGAAGGGCATCGACTTCAAGGAGGACGGCAACATCC
artmir-2 (1) ------CCCGCGCCGAGGTGAAGTTCGAGGGCGACACCCTGGTGAACCGCATCGAGCTGAAGGGCATCGACTTCAAGGAGGACGGCAACATCC
artmir-3 (1) ------CCCGCGCCGAGGTGAAGTTCGAGGGCGACACCCTGGTGAACCGCATCGAGCTGAAGGGCATCGACTTCAAGGAGGACGGCAACATCC
artmir-4 (1) ------CCCGCGCCGAGGTGAAGTTCGAGGGCGACACCCTGGTGAACCGCATCGAGCTGAAGGGCATCGACTTCAAGGAGGACGGCAACATCC
Section 2
(94) 94 100 110 120 130 140 150 160 170 186
EGFPm_X (94) TGGGGCACAAGCTGGAGTACAACTACAACAGCCACAACGTCTATATCATGGCCGACAAGCAGAAGAACGGCATCAAGGTGAACTTCAAGATCC
artmir-1 (88) TGGGGCACAAGCTGGAGTACAACTACAACAGCCACAACGTCTATATCATGGCCGACAAGCAGAAGAACGGCATCAAGGTGAACTTCAAGATCC
artmir-2 (88) TGGGGCACAAGCTGGAGTACAACTACAACAGCCACAACGTCTATATCATGGCCGACAAGCAGAAGAACGGCATCAAGGTGAACTTCAAGATCC
artmir-3 (88) TGGGGCACAAGCTGGAGTACAACTACAACAGCCACAACGTCTATATCATGGCCGACAAGCAGAAGAACGGCATCAAGGTGAACTTCAAGATCC
artmir-4 (88) TGGGGCACAAGCTGGAGTACAACTACAACAGCCACAACGTCTATATCATGGCCGACAAGCAGAAGAACGGCATCAAGGTGAACTTCAAGATCC
Section 3
(187) 187 EGFPm 200 exon 1 210 220 230 240 250 260 279
EGFPm_X (187) GCCACAACATCGAGGACGGCAGCGTGCAGNNNNNNNNNNNNNNNNNNNNNNNNNNNNNNNNNNNNNNNNNNNNNNNNNNNNNNNNNNNNNNNN
artmir-1 (181) GCCACAACATCGAGGACGGCAGCGTGCAG--
artmir-2 (181) GCCACAACATCGAGGACGGCAGCGTGCAG--
artmir-3 (181) GCCACAACATCGAGGACGGCAGCGTGCAG--
artmir-4 (181) GCCACAACATCGAGGACGGCAGCGTGCAG--
intron = mirtron
Section 4
(280) 280 290 300 310 320 330 340 357
EGFPm_X (280) NNNNNNNNNNNCTGGCCGACCACTACCAGCAGAACACCCCCATCGGCGACGGCCCCGTGCTGCTGCCCGACAACCACT
artmir-1 (210) -----------CTGGCCGACCACTACCAGCAGAACACCCCCATCGGCGACGGCCCCGTGCTGCTGCCCGACAACC---
artmir-2 (210) -----------CTGGCCGACCACTACCAGCAGAACACCCCCATCGGCGACGGCCCCGTGCTGCTGCCCGACAACC---
artmir-3 (210) -----------CTGGCCGACCACTACCAGCAGAACACCCCCATCGGCGACGGCCCCGTGCTGCTGCCCGACAACC---
artmir-4 (210) -----------CTGGCCGACCACTACCAGCAGAACACCCCCATCGGCGACGGCCCCGTGCTGCTGCCCGACAACC---
exon 2 EGFPm
