# Supplementary material for: Posttranscriptional Regulation of the Human ABCG2 Multidrug Transporter Protein by Artificial Mirtrons
Source: Genes (Basel). 2021 Jul 13;12(7):1068. doi: 10.3390/genes12071068 (PMC8307164; doi:10.3390/genes12071068)
Supplement: Supplementary file 1 [file genes-12-01068-s001.zip › Suppl_Figure_Legends.pdf]

Supplementary Figure S1. Predicted splicing parameters and structure of artificial mirtron 1 (art1)

(A) Sequence of art1 embedded in EGFPm sequence. EGFPm coding sequence is indicated by green letters, the loop region, which was originated from mmu-mir-1224 is shown by grey background, while the predicted branch point is shown by pink letter (inside the loop region).

(B) Splicing donor and acceptor site predictions using the Softberry program, highest scores indicating the best local motifs.

(C) Predicted secondary structure of art1.

Supplementary Figure S2. Predicted splicing parameters and structure of artificial mirtron 2 (art2)

(A) Sequence of art2 embedded in EGFPm sequence. EGFPm coding sequence is indicated by green letters, the loop region, which was originated from mmu-mir-1224 is shown by grey background, while the predicted branch point is shown by pink letter (inside the loop region).

(B) Splicing donor and acceptor site predictions using the Softberry program, highest scores indicating the best local motifs.

(C) Predicted secondary structure of art2.

Supplementary Figure S3. Predicted splicing parameters and structure of artificial mirtron 3 (art3)

(A) Sequence of art3 embedded in EGFPm sequence. EGFPm coding sequence is indicated by green letters, the loop region, which was originated from mmu-mir-1224 is shown by grey background, while the predicted branch point is shown by pink letter (inside the loop region).

(B) Splicing donor and acceptor site predictions using the Softberry program, highest scores indicating the best local motifs.

(C) Predicted secondary structure of art3. There are two potential secondary structures, one of them (left) is similar to art1, 2 and 4, while the other one (right) has a longer non-base paired stem region compared to the others.

Supplementary Figure S4. Predicted splicing parameters and structure of artificial mirtron 4 (art4)

(A) Sequence of art4 embedded in EGFPm sequence. EGFPm coding sequence is indicated by green letters, the loop region, which was originated from mmu-mir-1224 is shown by grey background, while the predicted branch point is shown by pink letter (inside the loop region).

(B) Splicing donor and acceptor site predictions using the Softberry program, highest scores indicating the best local motifs.

(C) Predicted secondary structure of art4.

Supplementary Figure S5. Microscopy images of untransfected HeLa cells

Representative microscopy images of HeLa cells at two different magnifications are shown. Cell nuclei were stained with Hoechst 33342 dye, brightfield and composite (“overlayed”) images are shown to indicate cell boundaries. To validate GFP signals after transfection, autofluorescence background is shown at excitation intensity ten times of what is applied on Figure 2B. The scale bar of 200 μm is placed on the overlayed image of 40x magnification.

Supplementary Figure S6. Sequence alignment of RT-PCR products of spliced mRNAs versus their unspliced form

Sequence alignment of RT-PCR products of spliced mirtron-containing EGFP mRNAs. EGFPm_X serves as reference sequence, where 'NNN' nucleotides represent the place of the respective spliced intron=mirtron (indicated by blue). 3'-end of the first exon of EGFPm is indicated by red line, while 5'-end of the second exon is indicated by green line.
